# A Total Internal Reflection Microscopy (TIRM)-Based Approach for Direct Characterization of Polymer Brush Conformational Change in Aqueous Solution

**DOI:** 10.1021/acsmacrolett.4c00476

**Published:** 2024-10-04

**Authors:** Jiahao Wu, Feng Cao, Pui Wo Felix Yeung, Manjia Li, Kohji Ohno, To Ngai

**Affiliations:** † Department of Chemistry, The Chinese University of Hong Kong, Shatin, N.T., Hong Kong 999077, China; ‡ Department of Chemical and Biological Engineering, The Hong Kong University of Science and Technology, Clear Water Bay, Kowloon, Hong Kong 999077, China; § Department of Materials Science, Graduate School of Engineering, Osaka Metropolitan University, Sakai, Osaka 599-8531, Japan

## Abstract

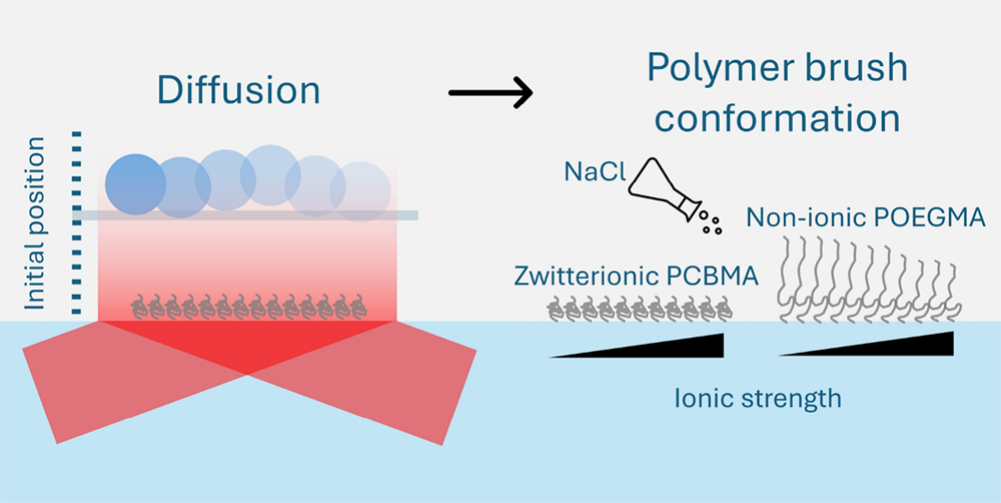

This study presents a novel approach utilizing total internal reflection microscopy (TIRM) to effectively characterize the swelling and collapse of polymer brushes in aqueous solutions. Zwitterionic poly(carboxybetaine methacrylate) (PCBMA) and nonionic poly[oligo(ethylene glycol) methyl ether methacrylate] (POEGMA) brushes are chosen as model systems. By investigation of an intriguing theory–experiment discrepancy observed during the measurement of near-wall hindered diffusion, valuable insights into the compressibility of polymer brushes are obtained, revealing their conformational information in aqueous solution. The results demonstrate that zwitterionic PCBMA brushes exhibit minimal antipolyelectrolyte effects in 0.1–10 mM NaCl solution but undergo significant swelling with increasing pH. On the other hand, nonionic POEGMA brushes exhibit similar responses to ionic strength as weak polyelectrolyte brushes. These unexpected findings enhance our understanding of polymer brushes beyond classical theories. The TIRM-based approach proves to be effective for characterizing polymer brushes and other soft nanomaterials.

Polymer brushes, thin surface coatings with polymer chains attached to a solid surface,^1^ are widely used to modify interface properties, driving advancements in surface and interface engineering.^2^ In recent years, stimuli-responsive brushes have seen rapid development. These brushes exhibit “smart” behavior by reversibly swelling and collapsing in response to external factors like pH, temperature, and ionic strength.^3^ This versatility makes them suitable for applications like switchable surfaces,^4^ adaptive coatings,^5^ membrane separations,^6^ and controlled drug delivery systems.^7^ With the increasing demand for stimuli-responsive materials, understanding their behavior under real-time conditions is crucial. The near-surface interaction and diffusion near polymer brushes also play key roles in advancing research by providing insights into the connection between the coating and the surrounding.

Existing analytical techniques like ellipsometry,^8^ atomic force microscopy (AFM),^9−11^ and small-angle X-ray scattering (SAXS)^12^ have been used to study the swelling and collapse of polymer brushes. While these methods have been valuable in many applications, they come with certain limitations that highlight the ongoing need for development in this area. For example, in ellipsometry, challenges might arise from the coupled determination of thickness and refractive index, especially in complex systems like heterogeneous polymer brushes with varying refractive indices during conformational changes.^13^ It is encouraging to note that researchers have presented some simulation works to address this issue by predicting the refractive index of swollen polymer brushes.^14^ Regarding AFM measurements, this technique is highly effective in visualizing surface morphology. However, determining conformation of polymer brushes using AFM typically requires the probe to identify the substrate, necessitating the stretching of samples to expose the substrate.^10^ Additionally, under high load conditions, the AFM probe can compress the brushes, resulting in an underestimation of film thickness.^11^ With effective scattering and careful analysis with appropriate models, SAXS provides insights into micro- or nanoscale structures of heterosystems, focusing on parameters like particle size, coating structure, and surface-area-to-volume ratios.^13^ But current applications of SAXS predominantly involve polymer brushes on nanoparticles rather than flat surfaces.^15^ We also find that existing characterization methods seldom establish a direct connection among the polymer conformation, near-surface diffusion, and near-surface interactions in the measurements.

Herein, we introduce a new approach using total internal reflection microscopy (TIRM) to investigate the swelling and collapse of polymer brushes. TIRM is an ultrasensitive surface force characterization technique capable of visualizing particle location at the nanometer scale and measuring near-surface interactions at the *k*_B_*T* energy level.^16^ There are a few prior studies using TIRM to measure the restructuring of polymer brushes at different ionic strengths, including zwitterionic PMPC, PMAPS, and nonionic PEO–PPO–PEO.^17,18^ Jumai’an et al. also investigate the specific ion effects (MgSO_4_ and NaCl) on these three types of polymer brushes, demonstrating the great potential of TIRM in this research area.^19^

However, prior TIRM studies usually need probes coated with the same polymer brushes as those grafted on the surface, and information about polymer brush conformation was obtained through steric repulsion fitting between the polymer-grafted probes and surface, akin to a “contact mode”. In contrast, our study aims to pioneer a “noncontact” mode by tracking the motion of free-moving particles above the surface using a high-frame-rate camera. We identified a discrepancy between two localization methods for these particles, linking near-surface diffusion with polymer brush conformation characterization. Our approach, while retaining the advantages of the technique, minimizes constraints on the probe surface and reduces reliance on steric interactions in the measurements. After examining the response to ionic strength of two common antifouling polymer brushes, zwitterionic poly(carboxybetaine methacrylate) (PCBMA) and nonionic poly[oligo(ethylene glycol) methyl ether methacrylate] (POEGMA), we find that the new approach offers superior precision in detecting changes in the outer soft regions of polymer brushes. Our novel findings on the ionic strength response of these brushes advance our understanding of this field beyond common predictions. Furthermore, given the widespread use of TIRM in surface force measurement, our study’s contributions enable future research to sensitively observe changes in surface status, near-surface diffusion, and interactions in a single measurement.

The first step of this new approach is the apply the excellent localization ability of TIRM to measure the vertical diffusion coefficient *D*_⊥_(*h*) near polymer-grafted surfaces using particle tracking and the Einstein–Smoluchowski equation (Scheme 1).^20^ What sets our work apart from previous studies is the utilization of a fast-frame-rate camera (∼400 fps), providing the necessary temporal resolution to directly measure this elevation-dependent diffusivity. Previous research has highlighted that an inadequate frame rate cannot ensure a Gaussian distribution of particle displacement within the evanescent wave area,^21^ which can cause errors when measuring the diffusion coefficient. By employing a sufficiently fast frame rate in our study, we ensure that the distribution of particle movement is Gaussian and that the mean square displacement (MSD) versus delta time relationship is linear during the calculations (Scheme 1d), so that the calculation is direct and correct.

**Scheme 1:**
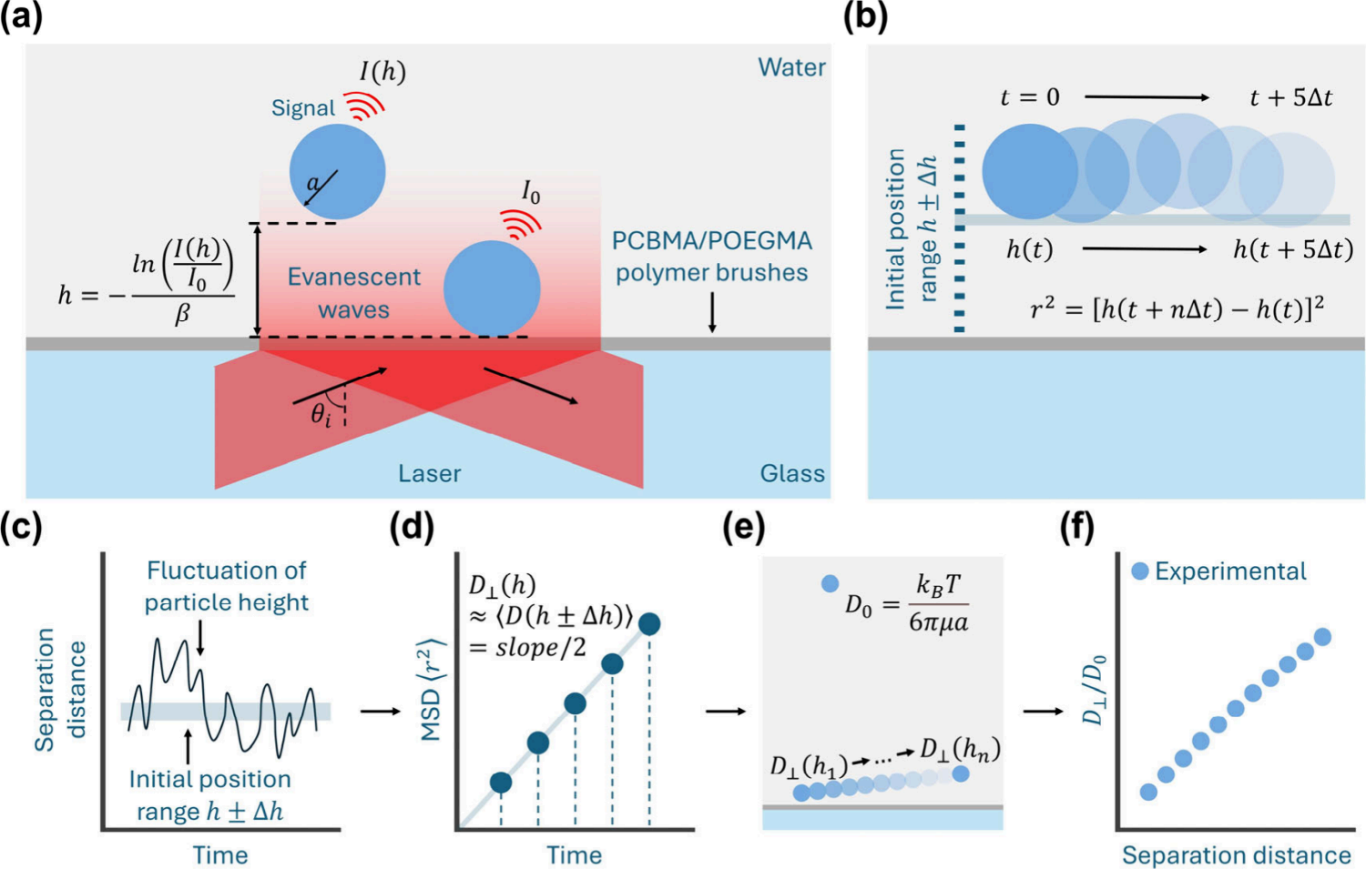
Schematic Illustration of the New Approach for Detecting Changes in Polymer Brushes (a) The principle of localizing the vertical position *h* of free-moving polystyrene tracers above a surface grafted with polymer brushes. (b) The method for collecting the squared displacement *r*^2^ of a free moving tracer, whose initial position of the bottom falls within the investigated range *h* ± Δ*h* (with Δ*h* set to 5 nm). The squared displacement data is collected after Δ*t*, 2Δ*t*, ···, and 5Δ*t*. The scale in the illustration does not represent the actual size. (c) To measure the diffusion coefficient at a certain height, all 5Δ*t* subunits from the particle’s height fluctuations are collected when the initial position of particle bottom falls within *h* ± Δ*h*. These sub-trajectories are used to calculate the mean squared displacement (MSD) after Δ*t*, 2Δ*t*, ···, and 5Δ*t*. The relationship between MSD and time is described by the Einstein–Smoluchowski equation (eq S3). (d) After linearly fitting the MSD data, the vertical diffusion coefficient *D*_⊥_ is calculated by dividing the slope by 2. (e) In this study, the vertical diffusion coefficient is measured stepwise from *D*_⊥_(*h*_1_) to *D*_⊥_(*h*_*n*_) with *h*_*i*+1_ – *h*_*i*_ = 10 nm and *h*_*n*_ – *h*_1_ = 100 nm. (f) After comparing *D*_⊥_(*h*) with *D*_0_, the relationship between *D*_⊥_(*h*)/*D*_0_ and separation distance *h* is plotted in the figures, which are the foundation for characterizing changes in polymer conformation in aqueous solution with TIRM.

Following the procedure outlined in Scheme 1, Figure 1 presents the experimental results along with theoretical curves illustrating the relationship between vertical diffusion coefficient *D*_⊥_ and particle–surface distance *h*. The theoretical *D*_⊥_–*h* relationship near a rigid/free surface was proposed by Brenner in 1961 and is described by eq 1 and eq 2.^22^

**Figure 1:**
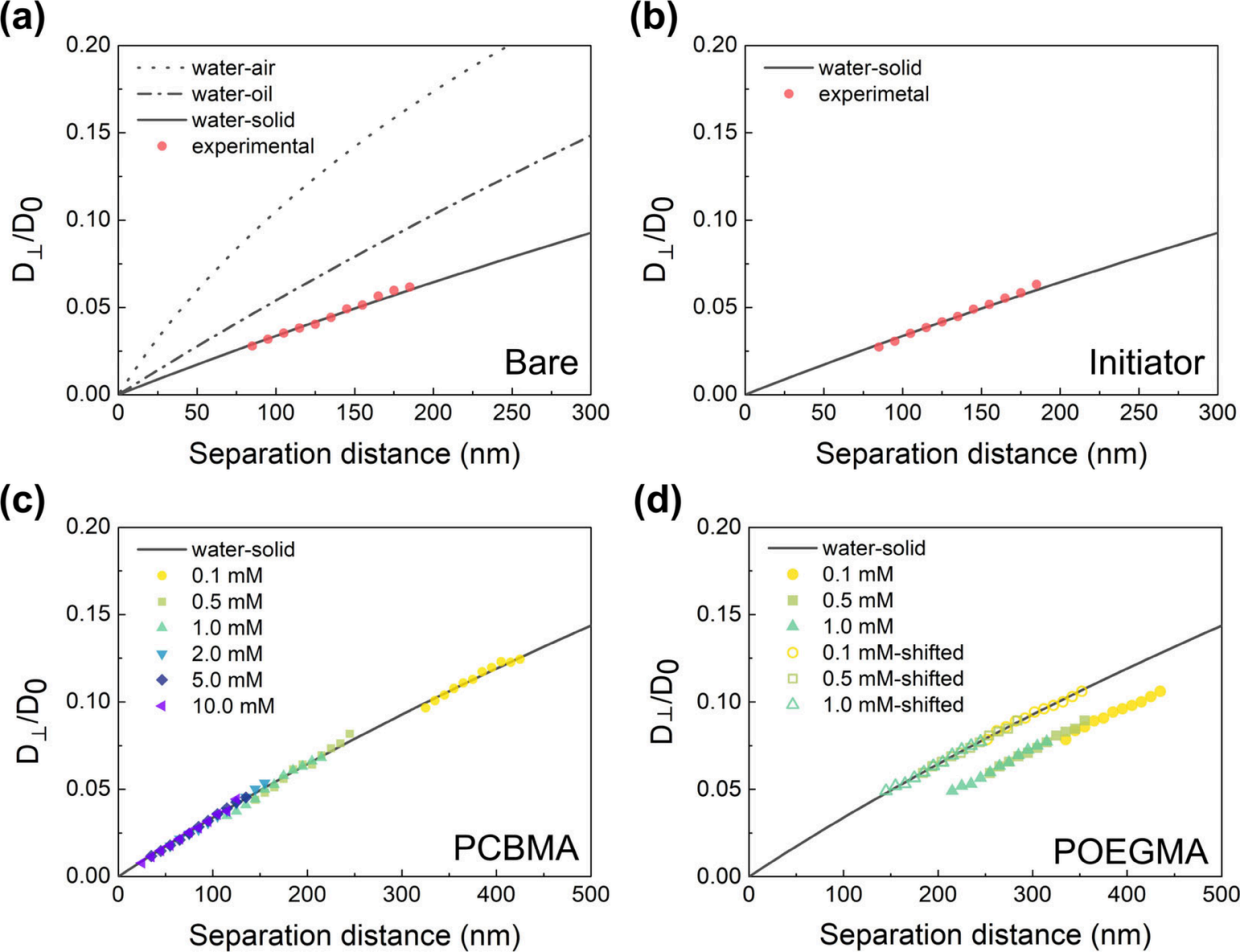
Relationship between the spatially resolved diffusion coefficient *D*_⊥_(*h*) and the separation distance to the surface for polystyrene tracers near a bare glass slide (a) and an initiator-grafted glass slide (b) in 1.0 mM NaCl solution, as well as a PCBMA-grafted glass slide (c) and a POEGMA-grafted glass slide (d) in NaCl solutions with different concentrations. The results are normalized to the bulk diffusion coefficient *D*_0_. The solid line and the dotted line represent Brenner’s theoretical prediction (eq 1 and eq 2) of the hindered diffusion near a rigid (e.g., water–solid) surface and a free (e.g., water–air) surface, respectively. The dash-dotted line is an empirical expectation for hindered diffusion near a water–oil interface, calculated by multiplying the *y*-axis values of the solid line by a factor of 1.6.^23^ The separation distance in the figures is determined by eq S2.

For hindered diffusion near a rigid surface:
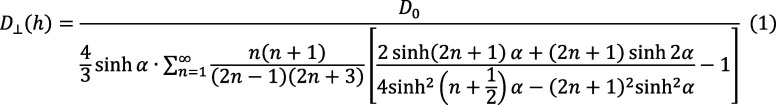
1For hindered diffusion near a free surface:
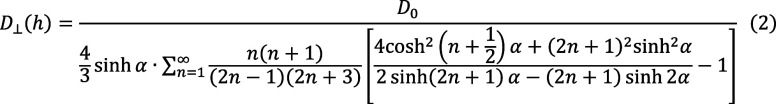
2where α = cosh^–1^(1 + *h*/*a*).

Generally, at the same particle–surface distance, the diffusion near a rigid surface, to which fluid adheres, is theoretically slower than the diffusion near a free surface (e.g., the water–air interface), on which the tangential stresses vanish. In other literature, the rigid and free surfaces are also known as the stick and slip boundary conditions, respectively.^23^ Apart from these two extreme conditions, the *D*_⊥_(*h*) curve near an oil–water interface (the dash-dotted line in Figure 1a) has been measured with TIRM by Helden and co-workers.^23^ In our case, we are wondering if the polymer brushes have a different hydrodynamic boundary condition when compared with an ideally rigid or free surface, and thereby influence the physics of the near-wall flow.^24^

For hindered diffusion near a bare surface (Figure 1a) and an initiator-grafted surface (Figure 1b), the good agreements between our results and the theoretical prediction indicate the reliability of our systems. For diffusion near a PCBMA-grafted surface (Figure 1c) or a POEGMA-grafted surface (Figure 1d), the slope of the experimental values still meets the theoretical prediction, indicating that the diffusion of contaminants does not show a remarkable change before they encounter the polymer brushes. Analysis with more details can be found in the SI. Interestingly, for the contaminants, the hydrodynamic edge of the polymer brush surfaces is rigid, but it does not mean that the polymer brushes are incompressible. In addition to the slope, it is worth noting that the gap between the theoretical prediction and the measured results offers us information about the compressibility of the grafted polymer brushes on the surfaces.

A systematic discussion by Oetama and Walz has indicated that the *D*_⊥_(*h*) curves could offer a trustworthy scale, in addition to the conventional optical way shown in Figure 1a, for determining the absolute particle–surface separation distance in TIRM measurements.^25^ The hydrodynamic approach becomes very useful, especially when the scattering intensity at *h* = 0 is hard to be measured.^23^ Moreover, an appealing early work done by Bevan and Prieve has pointed out that the deviations between different positioning methods in TIRM measurements had the potential to provide additional insight into the layer structure of adsorbed polymers.^26^ Therefore, we attempt to extend this idea and analyze the layer structure of both PCBMA and POEGMA brushes from the difference between the separation distance measured by the typical “optical” method and the more recent “hydrodynamic” approach simultaneously.

The theory–experiment deviation found in Figure 1 is caused by the compressibility beneath the hydrodynamic boundary of the polymer brushes. As shown in the schematic illustration in Figure 2a, a soft coating would be further compressed by the stuck polystyrene particles and thereby increase the value of *I*_0_ in eq S2, resulting in a larger *h*_optical_ than the *h*_hydro_ in measurements. The difference between the scales, Δ*h* = *h*_optical_ – *h*_hydro_, represents the extent to which the hydrodynamic edge of the coatings can be compressed by the stuck tracers. The hydrodynamic edge is defined as the point at which the separation distance in eq 1 and eq 2 reaches zero, corresponding to a value of *D*_⊥_(*h*) of zero as well. The measured *h*_optical_ and *h*_hydro_ values at each separation interval are taken together and considered as a single measurement of Δ*h* in a particular system.

**Figure 2:**
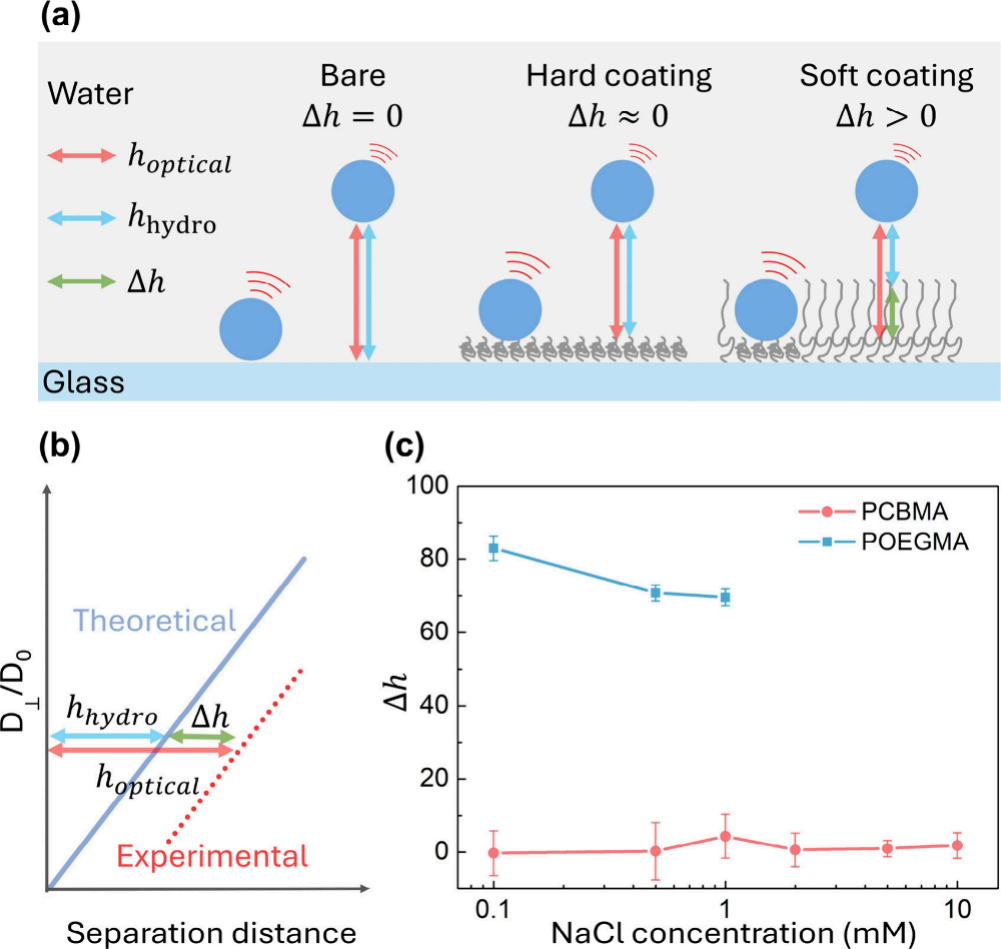
(a and b) Schematic illustrations of the approach of characterizing the layer structure of polymer brushes via Δ*h*, the difference between *h*_optical_ and *h*_hydro_. (c) The values of Δ*h* in measurements with PCBMA- or POEGMA-grafted glass slides at different ionic strengths.

Therefore, when measuring diffusion coefficient near a bare or initiator-grafted glass slide, it is reasonable that the values of Δ*h* were very close to zero (Table 1) due to their rigid hydrodynamic edges. As for the zwitterionic PCBMA brushes, the values of Δ*h* at different ionic strengths are shown in Figure 2c. Usually, zwitterionic polymer brushes are well-known for their antipolyelectrolyte effect, which is that the inter/intrachain interaction is screened and leads to the extension of polymer chains when more salt is added into the solutions.^27^ However, the values of Δ*h* for PCBMA brushes are nearly zero here and show no significant change with the 100-fold increase in salt concentration, indicating that the hydrodynamic boundary of the brushes is hard to further compress by the settled PS particles. We believe that the coincidence of the two scales was due to the insufficient swelling of the brushes. The AFM results shown in Figure S2 also prove that the thickness of the PCBMA polymer brushes in NaCl solutions is around 15 to 17 nm, which aligns closely with their dry state thickness.

**Table 1:** Values of Δ*h* in Measurements of Diffusion Coefficient near Different Substrates at Different Ionic Strengths

substrate	NaCl concentration (mM)	Δ*h* (nm)
bare glass surface	1.0	–1.6 ± 4.4
initiator-grafted surface	1.0	–1.5 ± 4.2
PCBMA-grafted surface	0.1	–0.3 ± 6.1
	0.5	0.3 ± 7.8
	1.0	4.3 ± 6.0
	2.0	0.6 ± 4.6
	5.0	1.0 ± 2.2
	10.0	1.8 ± 3.5
POEGMA-grafted surface	0.1	83.0 ± 3.4
	0.5	70.8 ± 2.2
	1.0	69.6 ± 2.3

The limited antipolyelectrolyte effect of PCBMA is not commonly observed in the field of zwitterionic brushes. Another reported example, demonstrated by Petroff et al., is zwitterionic PMPC polymer brushes that show no sensitivity to changes in ionic strength. Different to the collapsed PCBMA, PMPC was swollen in both low and high NaCl concentration solutions.^18^ They proposed that the layer thickness of adsorbed zwitterionic copolymers arises from a balance of intramolecular dipolar attraction and repulsion, which is possibly mediated by water solvation and specific zwitterionic moieties. From this perspective, the electrostatic intrachain dipole–dipole attraction maintains its dominant role in the PCBMA layer so that most of the polymer chains collapse and gather in the high-density region, forming a hydrodynamic boundary with a low softness. Another simulation work regarding CB polymers might offer a more direct explanation. Shao et al. calculated the partial charges of the cationic and anionic groups of zwitterionic moieties in CB polymers using quantum mechanical calculations and divided by their van der Waals volumes.^28^ The simulation suggested that CB moieties exhibit significant disparities in charge density between their cationic and anionic groups, resulting in limited associations.^28^ These limited associations lead to CB materials displaying inertness to external stimuli, contrary to the anticipated antipolyelectrolyte effects typically observed in zwitterionic materials.^27,29^ Our results agree with the simulations, which is quite encouraging as previous approaches for studying the structure–property aspects of zwitterionic brushes were mainly computational modeling and simulations while experimental characterization was considered as a challenging task.^27^

Meanwhile, the pH value could be another effective factor, rather than the salt concentration, to switch the layer structure of grafted PCBMA brushes. Therefore, we changed the pH value of the solutions from pH = 7 (0.1 mM NaCl solution) to pH = 10 (0.1 mM NaOH solution) and repeated the measurement (Figure 3). As shown in Table 2, the average Δ*h* increases from −0.3 to 38.3 nm. The larger Δ*h* indicates the swelling of PCBMA brushes at pH = 10 due to stronger deprotonation of the polymer chains. The overall electrostatic inter/intrachain interaction becomes repulsive, and a softer hydrodynamic boundary is formed by the swollen chains. This suggests that altering the pH value might be a more sensitive option than adding NaCl to switch the conformation and surface properties (e.g., antifouling, friction, and hydration) of zwitterionic polymer brushes within a range of low ionic strength, which is consistent with a recent simulation conducted by Miao et al.^30^

**Table 2:** Values of Δ*h* in Measurements near PCBMA-Grafted Glass Slides at Different pH Values^a^

substrate	Δ*h* (nm)
PCBMA-grafted, pH = 7	–0.3 ± 6.1
PCBMA-grafted, pH = 10	38.3 ± 3.9

a Measured in 0.1 mM NaCl solution and 0.1 mM NaOH solution, respectively.

**Figure 3:**
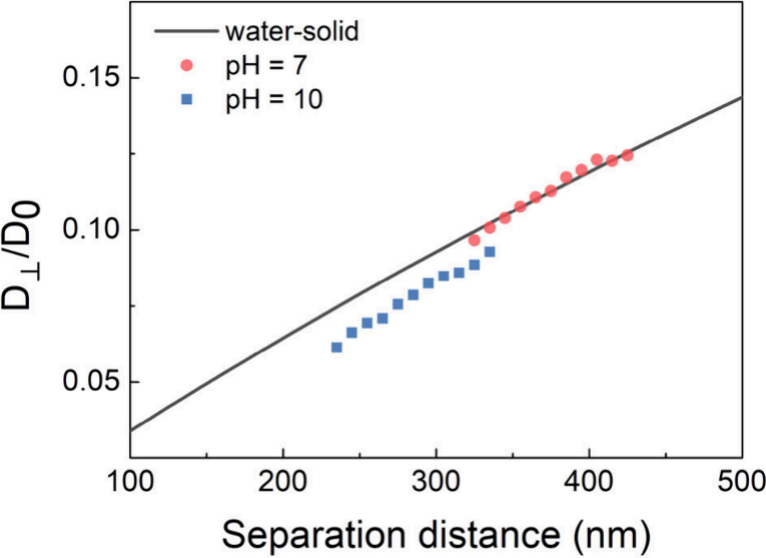
Relationship between the spatially resolved diffusion coefficient *D*_⊥_(*h*) and the particle–surface separation distance for the polystyrene tracers near a PCBMA-grafted glass slide at different pH values. The results are normalized to the bulk diffusion coefficient *D*_0_. The solid line represents Brenner’s theoretical prediction (eq 1) of the hindered diffusion of a spherical particle near a rigid surface.

As for the nonionic POEGMA brushes, the values of Δ*h* are drastically larger than those for PCBMA brushes. For instance, the measured Δ*h* for POEGMA brushes reaches 83.0 nm at an ionic strength of 0.1 mM, which illustrates a major difference between the conformation of PCBMA and POEGMA in NaCl solution. For zwitterionic PCBMA, attractive intrachain electrostatic interactions occur at low ionic strength. In contrast, POEGMA is nonionic, and its hydrophilic nature, along with the adsorption of hydroxide/chloride ions on the polymer chains, prevents strong intrachain attraction.^31^ This causes the POEGMA brushes to swell in the NaCl solution and form a hydrodynamic boundary with great softness.

When the salt concentration escalates from 0.1 to 1.0 mM, the Δ*h* value declines from 83.0 to 69.6 nm. Meanwhile, AFM measurements in Figure S2 indicate a change in swollen thickness from approximately 47 to 32 nm. Although the layer thickness measured by AFM is smaller than the TIRM results, the reduction in layer thickness is consistent with our findings. The disparity in swollen thickness can be attributed to the heterogeneous nature of the polymer brush layer, especially in the soft and loose outer region. Consequently, the AFM tip may easily compress the polymer brushes, resulting in an underestimation of the film thickness.

The weak polyelectrolyte effect exhibited by POEGMA can be attributed to two potential sources. First, hydroxide/chloride ion adsorption on the polymer chains contributes to this phenomenon.^31^ The reduction in ionic strength from 0.1, 0.5 to 1.0 mM, resulting in a decrease in the corresponding Debye length from 30.4, 13.6 to 9.8 nm, indicates a reduced range of electrostatic repulsion between the polymer chains and a similar trend of a decrease of Δ*h*. Another possible factor is the slight “salt-out” effect of chloride ions on POEGMA brushes.^32^ These highly hydrated ions disrupt the polymer–water hydrogen bond network and modify the surface tension of the polymer backbone, thereby destabilizing the polymer. However, achieving a ∼20% decrease in polymer brush thickness requires an increase in the range of chloride ionic strength from 0 to 500 mM, a range exceeding our case.^32^ Hence, the primary reason for the observed phenomenon in our study likely stems from ion adsorption on polymer chains, causing nonionic polymer brushes to exhibit responses to ionic strength akin to weak polyelectrolyte brushes.

In summary of this study, we employed a high-frame-rate camera to investigate near-wall hindered diffusion in the proximity of surfaces grafted with polymer brushes, utilizing the Einstein–Smoluchowski equation with exceptional accuracy and reliability. Additionally, we introduced a “noncontact” mode in TIRM-based polymer conformation characterization. The new method, utilizing free-moving probes with low kinetic energy, leverages the Δ*h* value to achieve precision in detecting alterations in the outer soft regions of polymer brushes while minimizing some previous constraints in characterization. Our findings unveiled that the antipolyelectrolyte effect of zwitterionic PCBMA was less significant than anticipated. In addition, our research highlighted a subtle polyelectrolyte effect in nonionic POEGMA in response to variations in ionic strength, emphasizing the need for further exploration in this area.

As for the limitations, the TIRM primarily operates near transparent substrates, such as the glass slides used in our study. However, TIRM can be adapted for use near various interfaces, such as oil–water interfaces or reflecting substrates coated with a gold layer.^23,33^ Future endeavors could involve extending this approach to diverse interface types. Moreover, the height limitation of polymer brushes is dictated by the depth of the evanescent wave area, typically spanning several hundred nanometers.

In conclusion, our study complements the characterization toolkit by providing valuable insights. The widespread applicability of TIRM in near-surface interaction investigations, combined with our study’s contributions, facilitates a holistic examination of polymer brush conformation, near-surface diffusion, and near-surface interactions in a single measurement. This integrated approach holds significant promise for informing the development of innovative polymer brushes in future research.

## Supporting Information Available

Experimental methods, information on data processing and boundary condition analysis, examples of MSD linear fitting, and AFM images of the polymer-grafted surfaces (PDF)
