# Supplementary material for: A Total Internal Reflection Microscopy (TIRM)-Based Approach for Direct Characterization of Polymer Brush Conformational Change in Aqueous Solution
Source: ACS Macro Lett. 2024 Oct 4;13(10):1376–82. doi: 10.1021/acsmacrolett.4c00476 (PMC11483946; doi:10.1021/acsmacrolett.4c00476)
Supplement: Supplementary file 1 — mz4c00476_si_001.pdf [file mz4c00476_si_001.pdf]

Supporting Information

A Total Internal Reflection Microscopy (TIRM)-Based Approach for Direct Characterization of Polymer Brush Conformational Change in Aqueous Solution

Jiahao Wu[a], Feng Cao[a], Pui Wo Felix Yeung[a], Manjia Li[b], Kohji Ohno[c], To Ngai[a]*

[a] *Department of Chemistry, The Chinese University of Hong Kong, Shatin, N.T., Hong Kong, China*

[b] *Department of Chemical and Biological Engineering, The Hong Kong University of Science and Technology, Clear Water Bay, Kowloon, Hong Kong, China*

[c] *Department of Materials Science, Graduate School of Engineering, Osaka Metropolitan University, Sakai, Osaka 599-8531, Japan*

*Corresponding author: Prof. To Ngai, email: tongai@cuhk.edu.hk

Experimental Methods

Materials

Sulfate polystyrene latex (Average diameter = 5.6 µm, ζ-potential = -100 mV, Invitrogen, USA), sodium chloride (Scharlau, Spain), hydrogen peroxide (30% v/v, Scharlau, Spain), sulfuric acid (98% v/v, RCI Labscan, Thailand), and ethanol (Duksan, South Korea) were utilized as received. Deionized water (18.2 MΩ·cm, Millipore, USA) was utilized for all experiments. Glass slides (BK-7, 75 mm × 25 mm, premium-grade, Fisher Scientific, USA) were sectioned into 25 mm × 25 mm pieces and subjected to an initial cleaning step, where they were immersed in Piranha solution (a 3:1 mixture of sulfuric acid and 30% hydrogen peroxide) at room temperature for 2 h. Following adequate rinsing with water and ethanol, the glass slides were dried using nitrogen gas and then placed in an ultraviolet-ozone plasma cleaner (PDC-002, Harrick Plasma, USA) for an additional 5-minute cleaning cycle prior to use. The brushes of PCBMA (Mn = 52,000 g/mol, graft density: 0.2 chains/nm^2) and POEGMA (Mn = 133,000 g/mol, graft density: 0.1 chains/nm^2) were grafted on the glass slides via surface-initiated atom transfer radical polymerization (SI-ATRP) according to our previous reports.[1] The dry thickness of the PCBMA and POEGMA, 13 nm and 18 nm, respectively, were measured by atomic force microscopy (XE-100, Park Systems, South Korea) in our previous study.[1]

Synthesis of PCBMA brushes

Carboxybetaine methacrylate (CBMA, 6.4 g) and ethyl 2-bromoisobutyrate (EBiB, ATRP free initiator, 5.4 mg) were dissolved in 2,2,2-trifluoroethanol (14 mL) and degassed with argon. The mixed solution was sent to the reaction vessel containing 2,2′-bipyridyl (Bpy, 18 mg), CuBr (8 mg), and the BPE-modified glass substrate under an argon atmosphere. The ATRP reaction was carried out for 24 h at 60 °C. After the reaction, the PCBMA brush-modified substrate was washed with methanol, ultrasonicated in methanol, repeatedly rinsed with methanol, water, and acetone, and finally dried by a flush of N_2 gas. The number-averaged molecular weight (M_n), weight-averaged molecular weight (M_w), and distribution of molecular weight (M_w/M_n) of free PCBMA produced from EBiB were determined by gel permeation chromatography (GPC, Shodex GPC-101, Tokyo, Japan) equipped with two columns (Shodex GPC SB-804), and a differential refractometer (Shodex RI-101). A mixed solution of water/acetonitrile = 3/2 containing 10 mM LiCl was used as the eluent at a flow rate of 0.8 mL/min. Poly(ethylene glycol) (PEG) standards were used to calibrate the GPC system. It is well-accepted that the values of M_n and M_w/M_n of free polymers are a good measure of those of the graft polymers.

Synthesis of POEGMA brushes

Oligo(ethylene glycol) methyl ether methacrylate (OEGMA, M_n = 500, 9.8 g) and EBiB (0.4 mg) were dissolved in anisole (10 mL) and degassed with argon. The mixed solution was sent to the reaction vessel containing 4,4’-dinonyl-2,2’-bipyridine (dNbipy, 94 mg), CuCl (43 mg), and the BPE-modified glass substrate under an argon atmosphere. The ATRP reaction was carried out for 20 h at 60 °C. After the reaction, the POEGMA brush-modified substrate was

washed with methanol, ultrasonicated in methanol, repeatedly rinsed with methanol, water, and acetone, and finally dried by a flush of N_2 gas. The M_n, M_w, and M_w/M_n of free POEGMA were determined by gel permeation chromatography (GPC, Shodex GPC-101, Tokyo, Japan) equipped with two columns (Shodex GPC KF-806L), and a differential refractometer (Shodex RI-101). *N,N*-Dimethylformamide (DMF) was used as the eluent at a flow rate of 0.8 mL/min. Poly(methyl methacrylate) (PMMA) standards were used to calibrate the GPC system.

Total internal reflection microscopy (TIRM)

As shown in Scheme 1, when a laser beam undergoes total internal reflection at a glass-water interface, evanescent waves will appear in the water near the interface and decay exponentially as the penetration distance increases. Meanwhile, the scattering from microspheres that enters the evanescent field has been found to have the same decay law:[2]

$$I(h) = I_0 e^{-\beta h}, \beta = \frac{4\pi}{\lambda}\sqrt{(n_1 sin\theta_i)^2 - n_2{}^2} (S1)$$

where n_1 and n_2 are the refractive index of glass and water separately; θ_i is the angle of incidence; h is the distance to the bottom surface; I_0 is the scattering intensity of stuck microspheres on the surface. Hence, the optical separation distance between a microsphere and the glass-water interface was obtained by comparing the real-time scattering intensity and its maximum.

$$h = -\frac{ln\left(\frac{I(h)}{I_0}\right)}{\beta} (S2)$$

During the measurements, sulfate latex dispersion was diluted to 0.8 mg/L with different concentrations of NaCl solution and injected into the flow cell at room temperature (298 K). At the bottom, a prism (70^0, BK-7) was attached to correct the angle of incidence for total internal reflection. The possible gap between the prism and the glass slide was immersed with a drop of immersion oil (Type F, n = 1.5180, Leica, Germany). Scattering signal from the near-surface particles was exerted by a laser beam generator (He-Ne, λ = 632.8 nm, 35 mW, Research Electro-Optics, USA) and assessed with an optical microscope (Olympus, Japan) and a high-speed sCMOS camera (Zyla, Andor, UK). 100,000 images (512 × 512, 16 bits) were taken at a rate of 418 frames per second to track a single particle. The zero point of the separation distance in the following discussion is determined by the scattering intensity of the tracers stuck on the bottom glass slides in a 100 mM NaCl solution. Finally, MATLAB (MathWorks, USA) codes were developed for image processing and data analysis.

Measuring diffusion coefficient with TIRM

The motion of a tracer along the vertical direction was divided into intervals according to the height, as the diffusion coefficient became distance-dependent in the near-wall region. Then, for tracers with Brownian motion, the vertical diffusion coefficient $D_\perp$ within each interval was calculated by the Einstein-Smoluchowski equation (Eqn. S3). Details regarding the principle and data processing could refer to our previous work.[3]

$$MSD = \langle [h(t+\Delta t) - h(t)]^2 \rangle = 2D_{\perp}(h)(S3)$$

Here, Δt was set to 2.393 ms, which was the fastest shutter duration of our camera. Five intervals of Δt were used for calculating the slope in Eqn. S3 and the vertical diffusion coefficient. As the size of pollutants varies in practice, the results were further normalized to the bulk diffusion coefficient, D_0, calculated by the Stoke-Einstein equation:

$$D_0 = \frac{k_B T}{6\pi\mu a}(S4)$$

where μ is the viscosity of the bulk solution, a is the diameter of the particles, k_B is the Boltzmann constant, and T is the room temperature in the measurements. To ensure the accuracy, i) the tracers were not subjected to strong electrostatic repulsion within the focused range so that the motion of the tracers over a short period was Brownian and applicable to the linear fitting of MSD, and ii) the data were collected in the height range where the potential energy was low to ensure sufficient data and enhance the accuracy of our measurements.[4]

Calculation of Δh

As illustrated in Scheme 1 and Figure 2(b), we first calculate our experimental diffusion coefficient at different separation distance. Then, as the $D_{\perp}(h) - h$ relationship is nearly linear within each height interval, we designate the midpoint of the interval as the $h_{optical}$ of corresponding $D_{\perp}(h)$. After obtaining a $D_{\perp}(h)$ value, we return to Brenner's prediction, the solid curves in the figures, to find out the corresponding value of h_{hydro}. The gap between $h_{optical}$ and h_{hydro} is Δh. We consider each Δh within a height interval as an individual data point. The average value of these data points corresponds to the Δh value presented in Table 1, with the standard deviation serving as the error bar.

Analysis of boundary conditions

To confirm the reliability of our system, including the guarantee of positioning accuracy and the elimination of external vibrations, we first examined the hindered diffusion near a bare glass surface. As shown in Figure 1(a), not only the slope but the absolute values of our results overlap Brenner's prediction for a rigid surface (the solid line in Figure 1(a)). This excellent agreement can be attributed to the reliability of our TIRM-based approach and the ultra-hydrophilic nature of the thoroughly cleaned glass slides, which promote water adhesion and satisfy the conditions for hindered diffusion near a rigid surface. We then replaced the bare glass slide with a glass slide immobilized with the initiator for ATRP, [(2-Bromo-2-methylpropionyl) oxy] propyl triethoxysilane (BPE) (Figure 1(b)). Although the surface chemistry of the glass slides had been partially altered, the effect of the initiator on the near-wall diffusion is imperceptible in TIRM measurements, indicating that the impact of the initiator can be excluded in the subsequent analysis.

Since the conformation of polymer brushes might be sensitive to ionic strength and thereby affect the near-wall diffusion, the measurements near PCBMA and POEGMA polymer brushes were performed in aqueous solutions with different NaCl concentrations. The morphology of the surfaces is characterized by AFM (Figure S2). As shown in Figure 1(c) and Figure 1(d),

the range of particle motion shifts to the substrate gradually with increasing ionic strength, hinting that the tracers were likely to be subjected to electrostatic repulsion from the bottom. We gradually moved down our range of measurement by increasing the salt concentration until the long-range repulsion could not balance the gravitational attraction (Figure 2(c) and 2(d)). The maximum NaCl concentration was 10 mM for PCBMA brushes and 1 mM for POEGMA brushes. In Figure 1(d), the data is also shifted to the left by some distance for a better comparison of the slope.

Interestingly, as shown in the Figure 1(c) and 1(d), even though surfaces grafted with polymer brushes seem to be softer than a bare glass surface, neither the zwitterionic PCBMA brushes nor the non-ionic POEGMA brushes exhibit obvious interference to the slope of the measured $D_{\perp}(h)$ curves at all ionic strengths we measured. In other words, the hydrodynamic boundary condition of the polymer brushes still meets the stick boundary condition for a rigid surface. The first layer of water molecules adheres to the shear plane of the polymer brushes tightly and is hard to slip, which indicates the well-known hydration layer around the anti-fouling polymer brushes. The results suggest that the diffusion of contaminants does not show a remarkable change before they encounter the polymer brushes. For the contaminants, the hydrodynamic edge of the polymer brush surfaces is rigid.

Atomic force microscopy (AFM)

AFM characterization was conducted using an AFM instrument (BioScope Resolve, Bruker) equipped with an open liquid cell. In a typical experiment, a polymer-modified glass substrate was positioned at the base of the liquid cell, which was then filled with a specific volume of various buffers. A DNP-10-A cantilever with a spring constant of 0.35 N/m was affixed to the liquid probe holder and fully submerged in the aqueous phase. The AFM tip was brought into proximity with the polymer-modified glass substrate.

A distinct pattern measuring 2 μm × 2 μm (width × length) was created using AFM scratching (Bruker, nanomanipulation mode) on the first sample immersed in a low salt concentration solution (0.1 mM NaCl). The scratching velocity in the x-y-z direction was set to 10 nm/s. The patterns generated were then visualized using contact mode AFM with a 0.2 V setpoint. Subsequently, higher salt concentration solutions (1.0 mM NaCl and 10.0 mM NaCl) were introduced to replace the initial buffer, with each buffer being left for approximately 1 hour. Images of the patterns under different conditions were captured using the AFM with identical parameters for each solution.

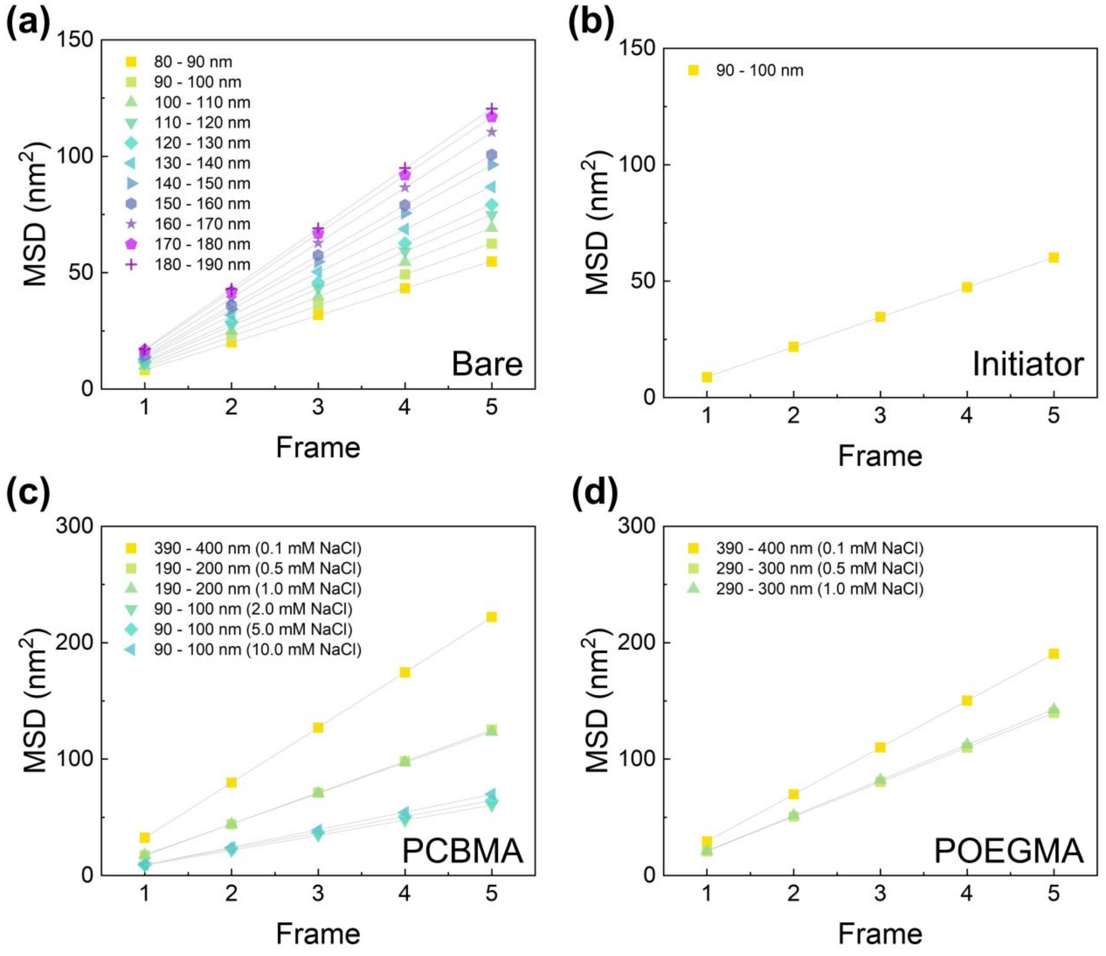

Figure S1 Linear fitting of MSD vs. Δt in various TIRM measurements: **(a)** bare surface, **(b)** initiator-grafted surface, **(c)** PCBMA-grafted surface, and **(d)** POEGMA surface. Figure S1(a) shows all MSD data points for the bare surface, while representative MSD data points for the other three groups are depicted in Figure S1(b), S1(c), and S1(d).

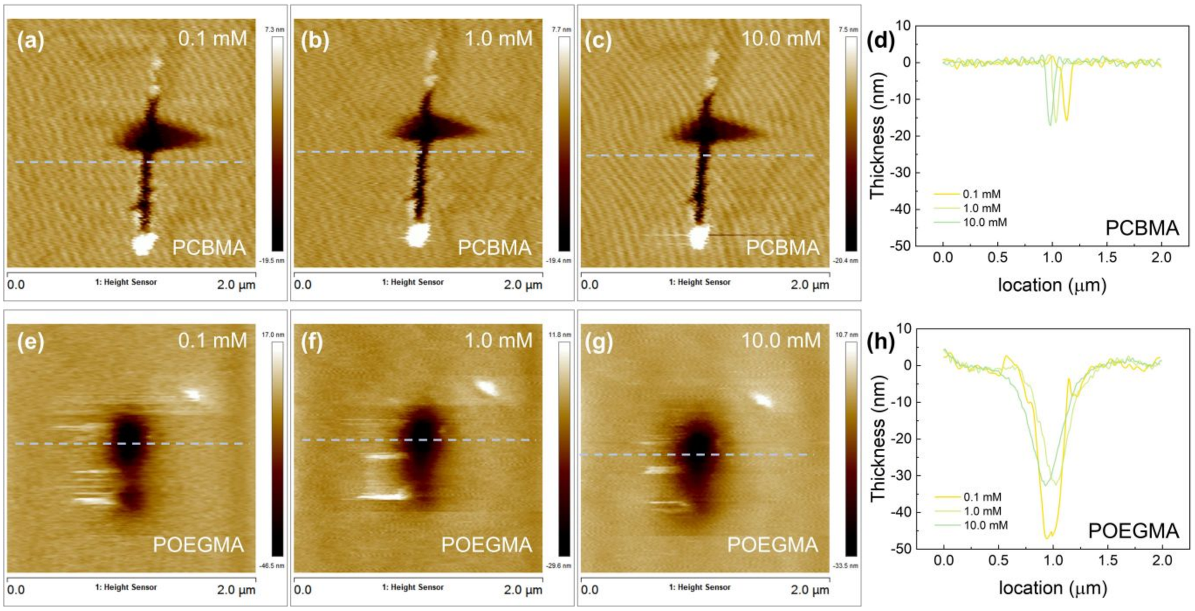

Figure S2 (a), (b), and (c) are AFM images of a 2 μm × 2 μm POEGMA-grafted surface with a stretched region in NaCl solution with concentration of 0.1 mM, 1.0 mM, and 10.0 mM, respectively. Panel (d) displays a cross-section along the dash lines in the AFM images, indicating the thickness of the PCBMA brushes at different ionic strengths. Similarly, (e), (f), and (g) are AFM images of a 2 μm × 2 μm POEGMA-grafted surface with a stretched region in NaCl solution with concentration of 0.1 mM, 1.0 mM, and 10.0 mM, respectively. Panel (h) displays a cross-section along the dash lines in the AFM images, indicating the thickness of the POEGMA brushes at different ionic strengths.

References

(1) Li, W.; Cao, F.; He, C.; Ohno, K.; Ngai, T. Measuring the Interactions between Protein-Coated Microspheres and Polymer Brushes in Aqueous Solutions. *Langmuir* **2018**, *34* (30), 8798-8806. DOI: 10.1021/acs.langmuir.8b01968.
(2) Prieve, D. C. Measurement of colloidal forces with TIRM. *Advances in Colloid and Interface Science* **1999**, *82* (1-3), 93-125.
(3) Cao, F.; Wu, J.; Li, Y.; Ngai, T. Measurements of Particle–Surface Interactions in Both Equilibrium and Nonequilibrium Systems. *Langmuir* **2019**, *35* (27), 8910-8920.
(4) Choi, C.; Margraves, C.; Kihm, K. Examination of near-wall hindered Brownian diffusion of nanoparticles: Experimental comparison to theories by Brenner (1961) and Goldman et al.(1967). *Physics of Fluids* **2007**, *19* (10), 103305.
